# Impact of Metformin Treatment on Human Placental Energy Production and Oxidative Stress

**DOI:** 10.3389/fcell.2022.935403

**Published:** 2022-06-17

**Authors:** Jane L. Tarry-Adkins, India G. Robinson, Rebecca M. Reynolds, Irving L. M. H. Aye, D. Stephen Charnock-Jones, Benjamin Jenkins, Albert Koulmann, Susan E. Ozanne, Catherine E. Aiken

**Affiliations:** ^1^ Department of Obstetrics and Gynaecology, The Rosie Hospital and NIHR Cambridge Biomedical Research Centre, University of Cambridge, Cambridge, United Kingdom; ^2^ Queen’s Medical Research Institute, Centre for Cardiovascular Science, University of Edinburgh, Edinburgh, United Kingdom; ^3^ Centre for Trophoblast Research, University of Cambridge, Cambridge, United Kingdom; ^4^ Wellcome-MRC Institute of Metabolic Science and Medical Research Council Metabolic Diseases Unit, Addenbrooke’s Hospital, University of Cambridge, Cambridge, United Kingdom

**Keywords:** placenta, metformin, trophoblast, oxidative stress, mitochondria, respiration, atp production, proton leak

## Abstract

Metformin is increasingly prescribed in pregnancy, with beneficial maternal effects. However, it is not known how metformin-treatment impacts metabolism and energy production in the developing feto-placental unit. We assessed the human placental response to metformin using both *in vivo* and *in vitro* treated samples. trophoblasts were derived from placentas collected from non-laboured Caesarean deliveries at term, then treated *in vitro* with metformin (0.01 mM, 0.1 mM or vehicle). Metformin-concentrations were measured using liquid-chromatography mass-spectrometry. Oxygen consumption in cultured-trophoblasts was measured using a Seahorse-XF Mito Stress Test. Markers of oxidative-stress were assayed using qRT-PCR. Metformin-transporter mRNA and protein-levels were determined by quantitative RT-PCR and Western-blotting respectively. Metformin concentrations were also measured in sample trios (maternal plasma/fetal plasma/placental tissue) from pregnancies exposed to metformin on clinical-grounds. Maternal and fetal metformin concentrations *in vivo* were highly correlated over a range of concentrations (R^2^ = 0.76, *p* < 0.001; average fetal:maternal ratio 1.5; range 0.8–2.1). Basal respiration in trophoblasts was reduced by metformin treatment (0.01 mM metformin; *p* < 0.05, 0.1 mM metformin; *p* < 0.001). Mitochondrial-dependent ATP production and proton leak were reduced after treatment with metformin (*p* < 0.001). Oxidative stress markers were significantly reduced in primary-trophoblast-cultures following treatment with metformin. There is a close linear relationship between placental, fetal, and maternal metformin concentrations. Primary-trophoblast cultures exposed to clinically-relevant metformin concentrations have reduced mitochondrial-respiration, mitochondrial-dependent ATP-production, and reduced markers of oxidative-stress. Given the crucial role of placental energy-production in supporting fetal growth and well-being during pregnancy, the implications of these findings are concerning for intrauterine fetal growth and longer-term metabolic programming in metformin-exposed pregnancies.

## 1 Introduction

Metformin is a biguanide drug with potential to impact cellular metabolism at multiple levels, including direct impacts on electron transport chain function, the mTOR pathway, and activation of AMP-activated protein kinase (Rena et al., 2017; Foretz et al., 2019). The organ-specific effects of metformin at various metabolically important sites, including the gut, liver, muscle, and adipose tissue, are increasingly understood (Stumvoll et al., 1995; Buse et al., 2016; Zabielski et al., 2017; Auger et al., 2019). However relatively little is known about potential impacts of metformin on the placenta, a site of critical importance in integrating maternal and fetal energy balance and metabolism.

The impact of metformin on the placenta is of escalating interest and concern as metformin is increasingly prescribed during pregnancy in various global settings (Priya and Kalra, 2018). Recent guidelines from multiple countries recommend metformin as a first-line drug treatment for gestational diabetes, which affects 5–30% of pregnant women worldwide (McIntyre et al., 2019). Metformin is relatively inexpensive and well-tolerated (Rowan et al., 2008), and thus suitable for use globally, including in low-resource settings. Metformin may have considerable benefits for maternal metabolic health during pregnancy, for example, limiting gestational weight gain and potentially reducing the risk of pre-eclampsia (Tarry-Adkins et al., 2021). However, concerns have been raised about the impact of metformin on the developing fetal-placental unit. Fetal exposure to metformin is associated with decreased birthweight followed by increased BMI and adiposity in mid-childhood, in both meta-analysis of human studies and animal models (Tarry-Adkins et al., 2019, 2020; Schoonejans et al., 2021; Hufnagel et al., 2022). This growth pattern of low birth-weight followed by post-natal catch-up growth has an established association with adverse programming of long-term cardio-metabolic outcomes (Kesavan and Devaskar, 2019). These findings raise important unexplored concerns about whether and how fetal-placental metabolism may be impacted by exposure to metformin.

A key determinant of fetal growth, and consequently metabolic programming, is placental energy production (Jansson and Powell, 2007). The developing fetus is dependent on the placenta (the interface with the maternal circulation) for oxygen delivery, nutrient transport, hormonal stimuli, and metabolic substrates (Burton and Fowden, 2015). In view of concerns regarding fetal growth and consequent long-term metabolic health in children exposed to metformin *in utero*, it is of key importance to understand as closely as possible the relationship between maternal, fetal, and placental metformin exposure, and thus how metformin might impact on placental cellular energy regulation. The placenta has a remarkably high basal energy requirement, which is met primarily *via* utilisation of glucose from the maternal circulation (Hay, 1991; Aye et al., 2020). This serves not only to support the bioenergetic needs of the placental tissue itself, which is highly metabolically active throughout pregnancy, but also to provide energy for the fetus. In other contexts and tissues, metformin is known to be an inhibitor of complex I of the electron transport chain (Foretz et al., 2019), therefore we hypothesise that metformin could prevent the placenta from meeting its high basal energy demand and hence compromise fetal well-being. Assessing the effect of metformin on energy production in the human placenta thus addresses an important knowledge gap.

The key aims of this study were therefore 1) to establish the relationship between maternal metformin administration during human pregnancy and feto-placental metformin exposure, and 2) to determine the impact of metformin exposure on placental energy production.

## 2 Materials and Methods

### 2.1 Human Tissue and Ethical Approvals

#### 2.1.1 Samples From Pregnancies Exposed to Metformin

*In vivo* experiments utilised samples from pregnancies where women were prescribed metformin during pregnancy on clinical grounds for gestational diabetes [500–2000 mg/day; demographic data Supplementary Table S1A]. Matched maternal plasma, umbilical cord plasma, and placenta samples from 10 pregnancies were obtained. The samples were collected following informed consent obtained by trained research midwives, who collected maternal and umbilical cord blood at birth. Samples were collected into EDTA, centrifuged within 1 h, the plasma separated, and stored at −80°C. Single placental biopsies were taken from the maternal side of the placenta and were stored in RNAlater solution (Qiagen, Manchester, United Kingdom) at 4°C for 24h, then frozen at −80°C. Measurement of metformin levels in these samples was conducted as detailed in section 2.4

#### 2.1.2 In Vitro Metformin-Treated Samples

Placenta were obtained from women undergoing elective Caesarean section at term [demographic data Supplementary Table S1B; inclusion and exclusion criteria Supplementary Table S2]. Placenta from healthy (non-diabetic donors) was used in order to remove any potential confounding factors of prior drug exposure or alterations in maternal metabolism. Informed consent was obtained by a research midwife, who subsequently collected the placenta immediately following birth. Fresh placentas were transferred to the laboratory within 5–10min of delivery for trophoblast isolation as described below.

#### 2.1.3 Ethical approvals

Study-specific ethical approval was granted under REC21/SC/0025 “The impact of metformin exposure on placenta ageing, metabolism, and mitochondrial function”. Stored maternal/neonatal/placental samples were kindly provided by the Edinburgh Reproductive Tissue Biobank (ERTBB; REC20/ES/0061). Fresh placental collections were obtained *via* the biobanks of the Centre for Trophoblast Research, Cambridge, United Kingdom (REC17/EE/0151) and from the Cambridge Blood and Stem Cell Biobank (REC18/EE/0199).

### 2.2 Primary Trophoblast Cultures

Cytotrophoblasts were isolated from freshly-collected whole term placental tissue, placental tissue as originally described by Kliman et al. (1986) with some modifications (Aye et al., 2013). Briefly, villous tissue was dissected free of decidua and blood vessels, mechanically minced, and digested three times in 0.25% trypsin and DNase I. The liberated cells were then separated over a discountinous Percoll (Sigma, Haverhill, United Kingdom) gradient (10%–70%) by centrifugation. Cells which migrated between the 35–55% Percoll layers were collected. An aliquot of cell suspension was stained with trypan blue (Thermo Fisher Scientific, Hemel Hempstead, United Kingdom) and counted using a Neubauer haemocytometer (Hawksley, Lancing, United Kingdom).

Cytotrophoblasts were cultured in complete media (BenchStable DMEM/F12, 10% fetal bovine serum; Supplementary Table S3A) and allowed to spontaneously differentiate into syncytiotrophoblast. Cells were plated at densities of: 2 million cells/well (for RT-PCR/protein quantification) or 1 million cells/well (for LC-MS). Cells were cultured at 37°C in 5%CO_2_ with media changed every 24 h. On day three, the cells were treated with either vehicle (dIH_2_0), 0.01 mM metformin, or 0.1 mM metformin (Sigma, Haverhill, United Kingdom). Cultured cells were checked daily under the microscope for viability and differentiation. Cells were plated at 0 h, then metformin-treated from 64 h to 88 h, with media changes every 24 h. Cells were harvested for storage or experiments at 88 h.

### 2.3 Assaying Respiratory Capacity in Trophoblasts

Oxygen consumption rate analyses were performed using the Mito Stress Test on an Agilent Seahorse XF-96 Extracellular Flux Analyser (Agilent Technologies, Cheadle, United Kingdom) according to the manufacturer’s instructions. Cells were plated on a Seahorse XF96 microplate at 0.5 million cells/well of a 96 well plate and maintained in culture as described above until 88 h when the mito stress test was performed. Before the assay, cell culture media was removed and replaced with freshly prepared XF media (Supplementary Table S3B) Mitochondrial modulators were loaded onto the sensor cartridge: port A; Oligomycin (2 µM), port B; Carbonyl cyanide-p- trifluoromethoxyphenyl-hydrazon (FCCP) (2 µM) and port C; Rotenone/Antimycin A (0.5 µM each). The analysis was run using 3 × 3min cycles with injection from sequential ports between each set. Data analysis was conducted using Wave Software (Agilent Technologies, Cheadle, United Kingdom).

### 2.4 Assaying Metformin Concentrations in Primary Trophoblasts, Placenta and Plasma

Metformin was isolated from plasma, cells, and tissue utilising an adapted version of a protocol previously described (Jenkins et al., 2020). Tissues were weighed and homogenised in a chloroform:methanol (2:1) solution using a Bioprep 24–1004 homogeniser (Allsheng, Hangzhou City, China) run at speed: 4.5 m/s, time: 30 s for 2 cycles. Deuterated 1,1-Dimethyl-d6-biguanide hydrochloride (metformin-d6 HCl; QMX laboratories, Thaxted, United Kingdom) was added as an internal standard to all samples, followed by further homogenisation. Samples were centrifuged and the aqueous layer concentrated, then reconstituted in chromatography starting condition buffer.

LC-MS analysis was performed using a Waters Acquity H-Class HPLC System (Waters, Wilmslow, United Kingdom) with the injection of the sample onto a Scherzo SM-C18 column (150 mm*3 mm I.D. 3 µM) maintained at 40^°^C. The mass spectrometer was a Thermo Scientific Exactive Orbitrap with a heated electrospray ionisation source (Thermo Fisher Scientific, Hemel Hempstead, United Kingdom). The mass spectrometer was calibrated immediately before sample analysis using positive and negative ionisation calibration solution. The metformin analysis was run in positive mode from 0 to 5min with scan rate set at 2Hz, giving a resolution of 50,000 (arbitrary units) with a full-scan range of m/z 100 to 200.

### 2.5 Gene Expression Quantification in Trophoblasts

RNA was extracted from 4 million syncytiotrophoblasts using an RNeasyPlus Mini Kit (Qiagen, Southampton, United Kingdom), following the manufacturer’s instructions. RNA quantification was performed using a Nanodrop spectrophotometer (Nanodrop Technologies, Wilmington, DE, United States). Agarose gels were run to confirm the integrity of the RNA. cDNA was synthesized using oligo-dT primers and M-MLV reverse transcriptase (Promega, Southampton, United Kingdom). Gene expression was determined using custom-designed primers (Merck, Hemel Hempstead, United Kingdom; Supplementary Table S4) and SYBR Green PCR master mix (Applied Biosystems, Warrington, United Kingdom) as described previously (Tarry-Adkins et al., 2009). Equal efficiency of the reverse transcription of RNA from all samples was confirmed through quantification of expression of the housekeeping gene *B2M,* expression of which did not differ with treatment.

### 2.6 Protein Quantification in Trophoblasts Using Western Blotting

Protein extraction and quantification was performed as previously described (Tarry-Adkins et al., 2015). 10% polyacrylamide gels were loaded (20ug protein) and analysed using densitometry. Antibodies to the following proteins were detected: Thiamine transporter-2 (13407-1-AP), serotonin transporter (19559-1-AP) (Proteintech, Manchester, United Kingdom) and norepinephrine transporter (NBP-1-60120) (Novus Biologicals, Cambridge, United Kingdom). Scanning, normalisation, and quantification of signals were performed as described previously (Tarry-Adkins et al., 2015). All secondary antibodies were anti-rabbit IgG horseradish-peroxidase-linked (Jackson Laboratories, Bar Harbor, ME, United States).

### 2.7 Mitochondrial DNA Copy Number in Trophoblasts

Total DNA (mitochondrial and nuclear) was extracted using phenol/chloroform, as previously described (Aiken et al., 2016). A ratiometric assay of a single-copy mitochondrial gene (cytochrome c oxidase; *COX1*), against a single-copy nuclear gene (insulin-like growth-factor-1 receptor; *IGF1R*) was used to estimate average copy number of mitochondrial DNA per cell. Real-time PCR using SYBR Green detection was performed as described previously (Aiken et al., 2016). Average mtDNA copy number per nuclear genome was calculated as 2 × 2^(ΔCT)^.

### 2.8 Statistical Analysis in Trophoblasts, Placental Tissue and Plasma

Data were analysed using hierarchal mixed linear regression models, with fixed effect for treatment group and random effect for placenta-of-origin. Demographic details were assessed as potential co-variates in the analysis (Supplementary Table S1A,B), however these were neither statistically significant nor improved the model fit, and were therefore not adjusted for in the final analysis. Modelling results are presented as beta values ± SEM. Numeric data are represented as means ± SEM. In graphical representations, box plots show median ± interquartile range (IQR), with whiskers representing 1.5xIQR. Where *p* values are reported, an alpha level <0.05 was pre-specified as statistically significant. Data analysis was conducted using R statistical software package v4.1.

## 3 Results

### 3.1 Correlation Between Maternal, Fetal, and Placental Metformin Concentrations

**FIGURE 1:**
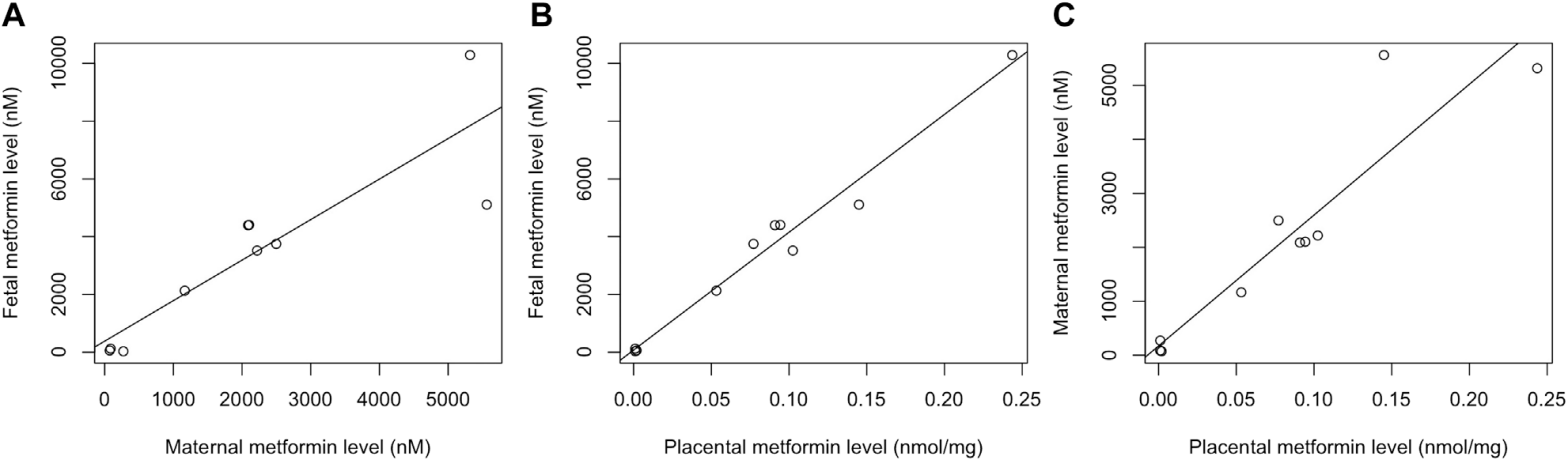
Metformin concentrations following *in vivo* administration with metformin during pregnancy. *N* = 10 simultaneously obtained sample trios: maternal plasma, umbilical cord plasma, and placental tissue. **(A)** Relationship between fetal and maternal plasma metformin concentrations, R^2^ = 0.84, *p* < 0.001; **(B)** Relationship between fetal plasma and placental metformin concentrations, R^2^ = 0.97, *p* < 0.001; **(C)** Relationship between maternal plasma and placental metformin concentrations, R^2^ = 0.76, *p* < 0.001.

Maternal and fetal metformin concentrations were correlated (R^2^ = 0.76, *p* < 0.001; Figure 1A, and were linear over a range of maternal plasma concentrations, with an average fetal:maternal ratio of 1.5 (range 0.8–2.1). Fetal and placental metformin concentrations were also highly correlated (R^2^ = 0.97, *p* < 0.001; Figure 1B within an *in vivo* fetal plasma concentration range that overlapped in the top of the range with the concentrations used in our primary cell culture model. Placental tissue metformin concentrations were also highly correlated with maternal circulating concentrations (R^2^ = 0.84, *p* < 0.001; Figure 1C, and this relationship remained linear over a range of circulating concentrations.

### 3.2 Metformin Concentrations in Cultured Primary Trophoblast and Placental Tissue

**FIGURE 2:**
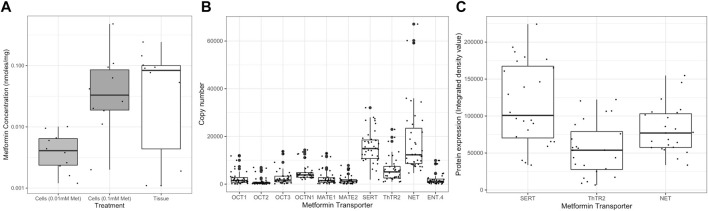
**(A)** Metformin concentration in trophoblasts treated *in vitro* with: (0.01mM and 0.1 mM) and placental tissue exposed *in vivo*. There was a non-significant difference between tissue metformin concentrations and metformin concentrations in 0.01 mM-treated cells (*p* = 0.08). There was no significant difference between tissue metformin concentrations and metformin concentrations in 0.1 mM-treated cells (*p* = 0.894). Metformin concentration is plotted on a log10 scale. Individual data points each represent a single trophoblast sample with points more than 1.5x IQR highlighted with larger dots. Light grey boxes: trophoblasts treated with 0.01 mM metformin; Dark grey boxes: 0.1 mM metformin; white boxes: Placental tissue from patients treated with metformin *in vivo*. **(B)** mRNA level of potential metformin transporters in trophoblasts (*n* = 30 samples). Genes assayed: *OCT-1* (SLC22A1), *OCT-2* (SLC22A2), *OCT-3* (SLC22A3), *MATE-1* (SLC47A1), *MATE-2* (SLC47A2), *SERT* (SLC6A4), *ThTR2* (SLC19A3), *NET* (SLC6A2) and *ENT-4* (SLC29A4). All copy numbers expressed relative to genomic DNA standard curve run on every plate. **(C)** Relative protein levels of SERT (SLC6A4), ThTR2 (SLC19A3), NET (SLC6A2) [All proteins were present at densities significantly above background (*p* < 0.05)].

Metformin enters primary trophoblast cultured *in vitro,* giving measured cellular concentrations in ranges over-lapping with those in *in vivo* exposed placentas (Figure 2A). No metformin was detectable in control samples. There was an approximately 10-fold difference between the metformin concentration in cultured primary trophoblasts treated with 0.01 mM metformin (median = 1.60mM, IQR 0.80–2.19mM, *n* = 10) versus 0.1 mM metformin (median = 15.03mM, IQR 6.30–25.13mM, *n* = 10), *p* < 0.001. Organic cation transporters that plausibly transport metformin (Liang and Giacomini, 2017) were present in variable amounts in trophoblasts (Figure 2B). High mRNA levels of serotonin transporter (*SERT*; SLC6A4), thiamine transporter 2 (*ThTR2*; SLC19A3), and norepinephrine transporter (*NET*; SLC6A2) relative to other organic cation transporters were observed, and these were confirmed *via* western blots (Figure 2C). These transporters have all been shown *via* immunohistochemistry to be present in trophoblast cell membranes on the maternal-facing aspect of the human placenta (Uhlen et al., 2015). Culture with metformin at either concentration did not affect the mRNA levels of any of the transporters compared to controls (data not shown).

### 3.3 Metformin Reduces Cellular Oxygen Consumption Rate in Trophoblasts

**FIGURE 3:**
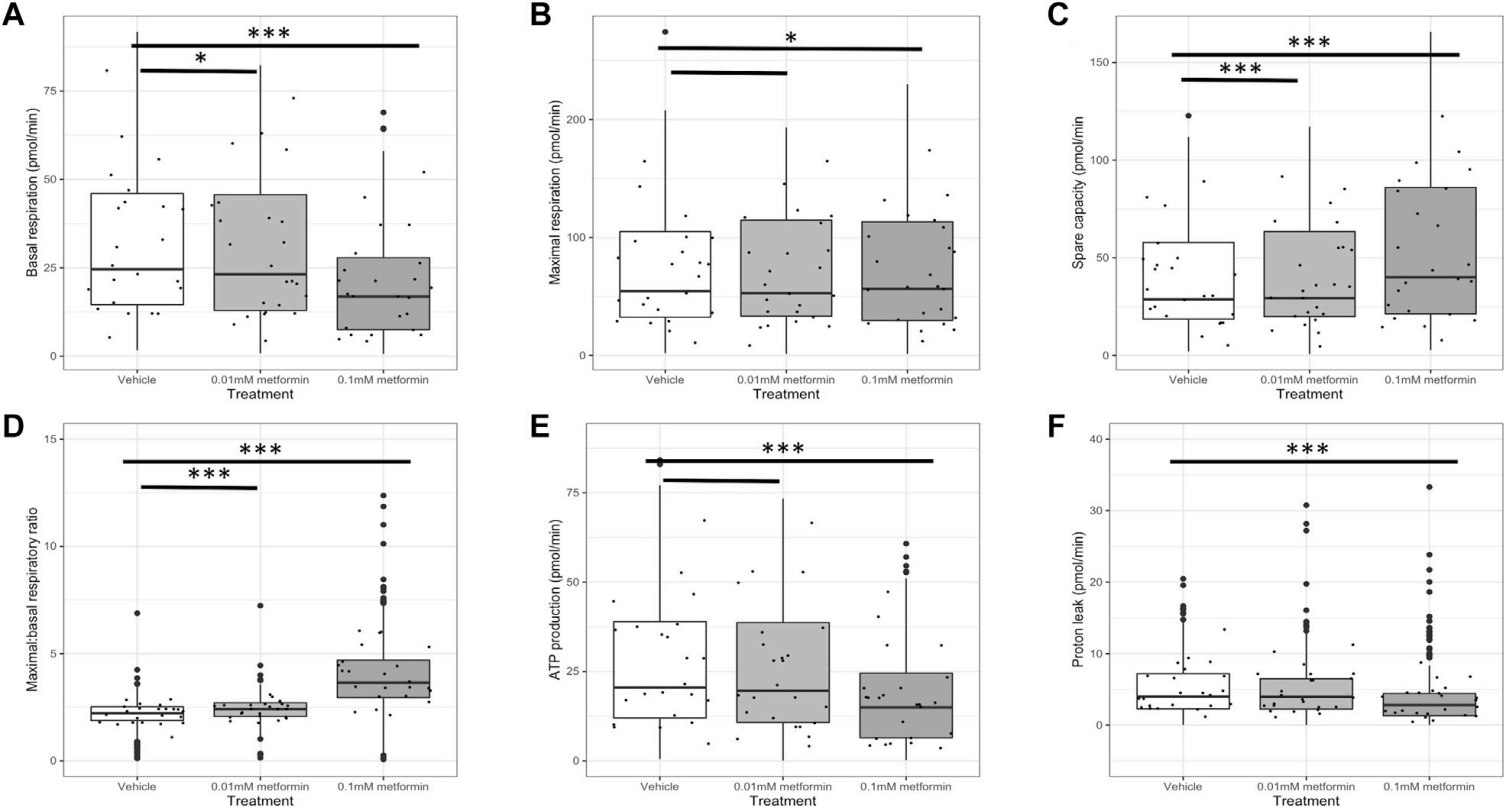
Respiratory parameters in trophoblasts: metformin (0.01 mM or 0.1 mM) versus control (*n* = 24 per group). Values are derived from parameter calculations comparing the oxygen consumption rate (OCR) under various conditions and expressed as pmol O_2_ consumed per minute. An example of the full stress test profiles obtained is presented as Supplementary Figure S1. **(A)** Basal respiration, defined as O_2_ consumption when respiring under normal conditions with non-mitochondrial O_2_ consumption subtracted; **(B)** Maximal respiration, defined as maximal respiratory rate measured following addition of an uncoupling agent with non-mitochondrial O_2_ consumption subtracted; **(C)** Spare respiratory capacity, defined as maximal respiration with basal respiration subtracted; **(D)** Ratio of maximal to basal respiratory capacity; **(E)** mitochondrial-dependent ATP production, defined as O_2_ consumption when respiring under normal conditions minus the rate after addition of an ATP synthase inhibitor; **(F)** Proton leak, defined as the minimal rate of respiration after addition of an ATP synthase inhibitor with non-mitochondrial O_2_ consumption subtracted. *p* values are derived from mixed hierarchal regression models with fixed effect for treatment groups and random effect for placenta-of-origin. ***p < 0.001, *p < 0.05, line only *p* < 0.1.

Basal respiration was reduced in trophoblasts in the presence of metformin compared to control (0.01 mM metformin; *p* < 0.05, 0.1 mM metformin; *p* < 0.001; Figure 3A. Maximum respiratory capacity was also reduced following culture with metformin (0.1 mM metformin *p* < 0.05; Figure 3B, although this did not reach statistical significance at a lower (0.01 mM) metformin concentration (*p* = 0.061). Spare respiratory capacity was significantly increased in the presence of metformin (0.01mM; *p* < 0.001, 0.1mM; *p* < 0.001; Figure 3C, as was the maximal:basal respiration ratio (0.01mM; *p* < 0.001, 0.1mM; *p* < 0.001; Figure 3D. ATP production and proton leak were both significantly reduced after culture with metformin 0.1 mM (*p* < 0.001), although these effects did not reach pre-specified statistical significance at a lower (0.01 mM) metformin concentration Figures 3E,F. There was no difference between experimental groups in the mtDNA copy number per cell Supplementary Figure S2A, or the RNAs encoding key regulators of mitochondrial biogenesis; *NRF1, NRF2, TFAM or PGC1a* Supplementary Figures S2B–E.

### 3.4 Metformin Decreases Cellular Oxidative Stress Markers in Trophoblasts

The expression of oxidative stress markers in trophoblasts was reduced following culture with metformin compared to control samples. Of the nine candidate genes selected for testing, there was a statistically significant reduction in expression for 4 of the genes (Table 1). Of the remaining genes, average copy number remained unchanged; in no case was there an increase in any marker of oxidative stress following treatment with metformin. There was a clear dose-dependency in the relationship between metformin exposure and oxidative stress. When 0.01 mM metformin was compared to controls, there were trends towards reduced expression of oxidative stress markers, although no gene met the pre-specified threshold for statistical significance. When 0.1 mM metformin was utilized, 4 out of 9 genes met the threshold for a statistically significant reduction in expression (Table 1).

**TABLE 1:** Relative mRNA level of oxidative stress genes in trophoblasts with metformin (0.01mM or 0.1mM, *n* = 27 per group). Copy number is expressed relative to the vehicle control group in all cases, and displayed as n ± SE. *p* values are derived from mixed-effects linear regression analysis with treatment group included as a fixed effect and placenta of origin as a random effect. *p* values that reached the pre-specified threshold for significance (<0.05) are displayed in bold.

Oxidative stress marker	0.01mM metformin	*p* Value	0.1mM metformin	*p* Value
Hif1a	−6048 (±3340)	0.076	−9838 (±3322)	**0.005**
Hif2a	−2371.04 (±11662)	0.840	−3014.60 (±11454)	0.797
MnSOD	−5645 (±13137)	0.670	−13459 (±12882)	0.304
IL6	18 (±117)	0.877	94 (±115)	0.417
XO	353 (±457)	0.442	−1395 (±464)	**0.004**
gp91phox	−1270 (±6911)	0.845	−20824 (±6890)	**0.004**
p47phox	−510 (±1573)	0.747	−1032 (±1585)	0.518
p22phox	−10806 (±5720)	0.065	−11652 (±5644)	**0.044**
P67phox	−242 (±2932.21)	0.934	−1110 (±2934)	0.707

The *p* values listed in bold refer to *p* values that reached the pre-specified threshold for significance (<0.05) (i.e., Hif1a; 0.005, XO; 0.004, gp91phox; 0.004 and p22phox; 0.0440).

## 4 Discussion

In view of the increasing numbers of pregnant women globally treated with metformin during pregnancy, it is essential to understand its impact on placental energy production which could have important consequences for fetal growth. In this study we demonstrate for the first time that exposure to concentrations of metformin similar to those in treated pregnant women reduces basal mitochondrial respiration in trophoblasts in a dose-dependent manner. Metformin-treated trophoblasts show a reduction in ATP production, despite maintaining good integrity as suggested by reduced proton leak. In keeping with reduced mitochondrial respiration in trophoblasts exposed to metformin, we also demonstrate a reduction in oxidative stress at gene expression level. These findings have important potential implications when considering the clinical use of metformin during pregnancy.

In other tissues and disease states, evidence suggests that metformin may be a direct inhibitor of complex I of the electron transport chain (Owen et al., 2000). However there may also be other pathways by which metformin impacts cellular energy production including inhibition of complex IV (LaMoia et al., 2022) or the lysosomal pathway *via* PEN2 (Ma et al., 2022). These mechanisms have not previously been explored in in human trophoblasts, which is an important target for future work. However our results are consistent with inhibition of electron transport chain activity, as has been reported previously in other rapidly dividing cells (Wheaton et al., 2014). Previous indirect evidence also implies that there may be electron transport chain inhibition with supra-physiological concentrations of metformin applied to primary villous cytotrophoblast (Brownfoot et al., 2016). In placental tissue a reduction in basal respiration is likely to be detrimental to physiological function, which relies on extensive cellular energy production that may exceed 2.5 kg ATP daily in the full-term placenta (Bax and Bloxam, 1997; Aye et al., 2020). High placental energy demands are driven by multiple processes necessary to support rapid fetal growth, including nutrient transport and protein synthesis, which alone may consume more than half of placental energy production (Schneider, 2000). Placental tissue must also fulfil its own bioenergetic needs to keep pace with fetal growth, and biosynthetic functions to co-ordinate maternal physiological adaptation to pregnancy, primarily *via* hormone synthesis [reviewed in (Aye et al., 2020)]. The impact of metformin to reduce basal respiration and ATP synthesis at clinically-relevant doses may thus have important impacts on placental function and raises concern regarding maintaining fetal growth. This is in keeping with our previous meta-analyses, which show that babies born to mothers with gestational diabetes who were exposed to metformin *in utero* are smaller at birth than their counterparts whose mothers received alternative therapies (Tarry-Adkins et al., 2020). Moreover, insufficient placental respiration and ATP production raises the possibility of placental insufficiency, which in extreme cases is a cause of spontaneous preterm birth (Morgan, 2016) and stillbirth (Silver, 2018). At present there is no clear evidence that metformin influences these outcomes from human clinical trials, but most studies are not powered for these relatively rare outcomes.

The risk versus benefit ratio of metformin therapy during pregnancy may be altered according to circumstances, particularly in high-risk pregnancies such as those affected by severe pre-eclampsia. Pre-eclampsia originates early in gestation, with incomplete conversion of the spiral arteries (Aplin et al., 2020), but manifests clinically much later in gestation, when a maternal systemic response including high blood pressure, proteinuria, and nervous system pre-excitation emerges (Chappell et al., 2021). This response is due to maternal systemic endothelial dysfunction driven by oxidative stress and pro-inflammatory factors from the placenta (Chappell et al., 2021). Placental metabolism is increasingly dependent on oxidative phosphorylation after the first trimester, which becomes less efficient even in healthy pregnancy as gestation progresses (Holland et al., 2017). In severe pre-eclampsia there is dysregulation of placental bioenergetics and biosynthetic homeostasis associated with altered mitochondrial structure and function (Marin et al., 2020). This results in inefficient electron transport chain activity in the placenta, which in turn leads to the high reactive oxygen species (ROS) generation (Roberts and Escudero, 2012) and reduced ATP production characteristic of accelerating severe pre-eclampsia (Marin et al., 2020). Our results show a significant reduction in mitochondrial respiration and proton leak in trophoblasts treated with metformin, which is a novel finding in human trophoblast, corresponding with a decrease in expression of markers of oxidative stress. This may be a mechanism by which metformin could be protective against the late maternal systemic effects of pre-eclampsia. By limiting oxidative phosphorylation and therefore preventing an accelerating cycle of ROS generation, the phenotype of severe pre-eclampsia may be ameliorated and pregnancy prolonged. This is in keeping with recent human trial results, showing trends towards later iatrogenic delivery in severe pre-eclampsia treated with metformin versus placebo (Cluver et al., 2021). There is therefore potential for beneficial effects of metformin in the context of severe preterm pre-eclampsia, which may be life-threatening for mothers and babies.

Our study has significant methodological advantages, including the availability of a relatively large and carefully-phenotyped cohort of non-laboured placentas from women who met clear inclusion and exclusion criteria. We also provide important confirmation of the therapeutic relevance of the concentrations of metformin used in our study. This increases the clinical relevance of our results and provides proof-of-principle of how trophoblasts are likely to respond *in vivo* to similar metformin exposure. We also show clear evidence that trophoblast concentrations of metformin *in vivo* correspond directly to those in maternal plasma, which is important in further establishing real-life relevance of our assays and results. We also recognise some limitations in our study. Although we establish evidence of reduced oxidative phosphorylation in metformin-treated trophoblast, our current methodology is unable to interrogate the specific site of electron transport chain blockade with metformin treatment and we have not directly shown a reduction in oxidative stress. This is an important focus of future research. A further future aim is to understand the consequences of reduced placental energy production for the fetus, which is not possible in our current primary cell model system, but amenable to investigation in animal models (Schoonejans et al., 2021; Hufnagel et al., 2022).

We conclude that primary human trophoblasts exposed to metformin in culture at clinically-relevant concentrations have reduced levels of mitochondrial respiration, cellular ATP production, and reduced cellular markers of oxidative stress. Given the crucial role of placental energy production in supporting fetal growth and well-being during pregnancy, these results are of key importance in refining assessment of the risk versus benefit ratio of clinical application of metformin in pregnancy.

## Data Availability

The original contributions presented in the study are included in the article/Supplementary Material, further inquiries can be directed to the corresponding author.

## Supplementary Material

Supplementary Figure S2 Mitochondrial biogenesis markers in trophoblasts: with metformin (0.01mM or 0.1mM) versus control. **(A)** Mitochondrial DNA copy number per cell (*n* = 17 per group); **(B)** Relative mRNA level of nuclear receptor factor-1 (*NRF1)* (*n* = 20 per group); **(C)** Relative mRNA level of nuclear receptor factor-2 (*NRF2)* (*n* = 20 per group); **(D)** Relative mRNA level of Mitochondrial transcription factor A (*TFAM)* (*n* = 20 per group); **(E)** Relative mRNA level of Peroxisome proliferator-activated receptor-gamma coactivator (PGC1α) (*n* = 20 per group). Individual data points each represent a single trophoblast sample with points more than 1.5x IQR highlighted with larger dots.

## Abbreviations

ATP, adenosine triphosphate; MATE-1 or, solute carrier family 47 member 1; OCR, Oxidative capacity rate = Organic transporter protein-1; OCT-1 or solute carrier family 22 member 1, SLC22A1 = Organic transporter protein-2 = OCT-2 or solute carrier family 22 member 2; OCTN1 or, Multidrug and toxin extrusion protein 1; SLC22A2, Organic transporter protein-3 = OCT-3 or solute carrier family 22 member 3; SLC22A3, Organic cation transporter; novel, type 1; SLC47A1, Multidrug and toxin extrusion protein 2 = MATE-2 or solute carrier family 47 member 2; SLC47A2, Norepinephrine transporter = NET or solute carrier family 6 member 2; SLC6A2, Thiamine transporter 2 = ThTr-2 or solute carrier family 19 member 3; SLC19A3, Serotonin transporter receptor = SERT or solute carrier family 6 member; SLC6A4, Equilibrative nucleoside transporter 4 = ENT4 or solute carrier family 29 member 4 = SLC29A4.
