# Supplementary figures and images for: Impact of Metformin Treatment on Human Placental Energy Production and Oxidative Stress
Source: Front Cell Dev Biol. 2022 Jun 17;10:935403. doi: 10.3389/fcell.2022.935403 (PMC9247405; doi:10.3389/fcell.2022.935403)

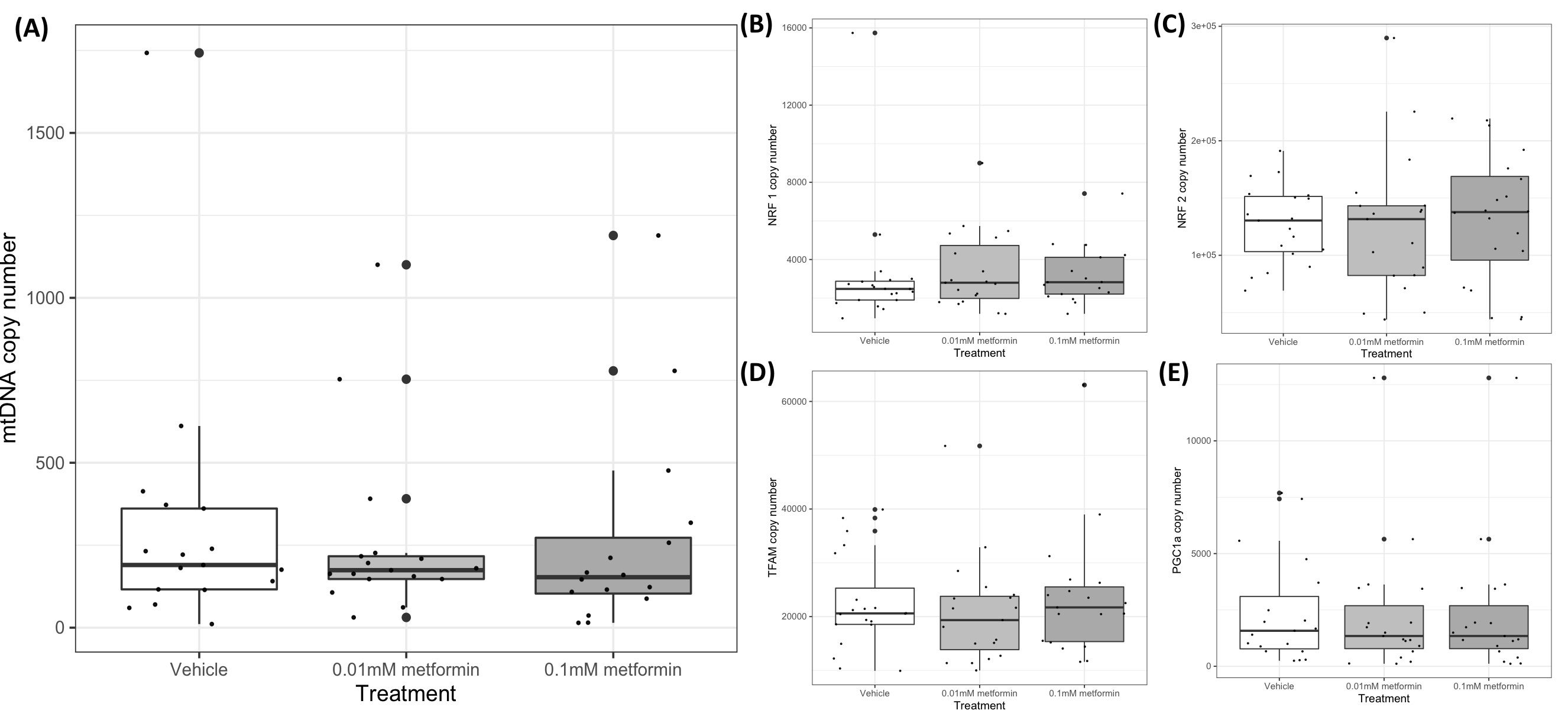

Supplement: Supplementary file 1 [file Image2.jpg]
