# Supplementary material for: Impact of Metformin Treatment on Human Placental Energy Production and Oxidative Stress
Source: Front Cell Dev Biol. 2022 Jun 17;10:935403. doi: 10.3389/fcell.2022.935403 (PMC9247405; doi:10.3389/fcell.2022.935403)
Supplement: Supplementary file 2 [file Table1.docx]

Supplementary Table 1: Demographic characteristics of study participants **A)** Pregnant women treated *in vivo* with metformin during pregnancy (n=10) **B)** Pregnant women donating placentas at elective Caesarean section for *in vitro* treatment with metformin (n=32)

**A**

| **Variable** | | **Mean (IQR) or N (%)** |
| --- | --- | --- |
| Maternal age (years) | | 33 (29 – 39) |
| Maternal BMI | | 44.61 (24.7 – 57.9) |
| Maternal weight category | BMI ≤25 | 1 (10%) |
|  | BMI >25 | 9 (90%) |
| Sex of baby | Female | 2 (20%) |
|  | Male | 8 (80%) |
| Placental weight (g) | | Weights not available |
| Birth weight (g) | | 3777 (2730 – 4750) |
| Birth weight centile | | 72.4 (57.5-89.7) |

| **Variable** | | **Mean (IQR) or N (%)** |
| --- | --- | --- |
| Maternal age (years) | | 34.8 (31.8 – 39.0) |
| Maternal BMI | | 27.41 (24.2 – 30.4) |
| Maternal weight category | BMI ≤25 | 11 (34%) |
|  | BMI >25 | 21 (66%) |
| Sex of baby | Female | 19 (59%) |
|  | Male | 13 (41%) |
| Placental weight (g) | | 613.5 (535.5 – 680.2) |
| Birth weight (g) | | 3558 (3228 – 3790) |
| Birth weight centile | | 72.4 (57.5-89.7) |

**B**
