# Supplementary material for: Impact of Metformin Treatment on Human Placental Energy Production and Oxidative Stress
Source: Front Cell Dev Biol. 2022 Jun 17;10:935403. doi: 10.3389/fcell.2022.935403 (PMC9247405; doi:10.3389/fcell.2022.935403)
Supplement: Supplementary file 3 [file Table2.docx]

Supplementary Table 2: List of inclusion and exclusion criteria for placental collections for isolation of primary trophoblast.

| Inclusion criteria | Exclusion criteria |
| --- | --- |
| Caesarean section with no prior labour | Multiple pregnancy |
|  | Known major fetal anomaly |
|  | Severe pre-eclampsia |
|  | Any form of diabetes in pregnancy |
|  | <37 weeks gestation |
|  | Treatment with metformin during pregnancy |
