# Supplementary material for: Impact of Metformin Treatment on Human Placental Energy Production and Oxidative Stress
Source: Front Cell Dev Biol. 2022 Jun 17;10:935403. doi: 10.3389/fcell.2022.935403 (PMC9247405; doi:10.3389/fcell.2022.935403)
Supplement: Supplementary file 4 [file Table3.docx]

Supplementary Table 3 A: Cell Media Composition

| Components |
| --- |
| BenchStable DMEM/F12 (1:1) with GlutaMax containing:  sodium bicarbonate (29mM), D-glucose (17.5mM), sodium pyruvate (0.5mM); (Cat no: A41920-01) |
| FBS (10% vol/vol) |
| Penicillin-Glutamine-Streptomycin (1% vol/vol) |
| Amphotericin B (0.1% vol/vol) |
| Gentamycin (0.1% vol/vol) |

Supplementary Table 3 B: Seahorse XF Media Composition

| Components | Stock conc | Volume (ml) | Working conc |
| --- | --- | --- | --- |
| XF Base media | - | 38.5 | N/A |
| Glucose | 2500mM | 0.5 | 10mM |
| Sodium pyruvate | 100mM | 0.5 | 1mM |
| L-glutamine | 200mM | 0.5 | 2mM |
| pH adjustment to 7.4 & H_2_0 top up | - | 1.5 | N/A |
