# Supplementary material for: Impact of Metformin Treatment on Human Placental Energy Production and Oxidative Stress
Source: Front Cell Dev Biol. 2022 Jun 17;10:935403. doi: 10.3389/fcell.2022.935403 (PMC9247405; doi:10.3389/fcell.2022.935403)
Supplement: Supplementary file 5 [file Table4.docx]

| **Primer** | **Sequence (F, 5’-3’)** | **Sequence (R, 5’-3’)** | **Product size (bp)** |
| --- | --- | --- | --- |
| ***SLC22A1 (OCT1)*** | TCTTCCTGGGTTTCACACCT | TAGCCAGGGGGTCTACACAG | 204 |
| ***SLC22A2 (OCT2)*** | TTCCTCTTGGCTCTGCTCTC | CTCTGGTTCCAGTCCACCTC | 227 |
| ***SLC22A3 (OCT3)*** | CACAAGCGCAGTGGTGTATC | CCCCTGCCACTATATTGCTT | 179 |
| ***SLC22A4 (OCTN1)*** | GCCTCATCTTCTTCCTGCTC | GCAGCGGGACACTGTTGT | 208 |
| ***SLC47A1-206 (MATE1)*** | CGAACGATGTTGGAAAGACA | CCCCACCAGCAAGATTAAGA | 164 |
| ***SLC47A2-206 (MATE2)*** | AGTTCCCCTGGCATTACCTT | CTAGGATCCTGACCGTGAGC | 196 |
| ***SLC6A4 (SERT)*** | GGATTGGTTATGCCATCTG | GAGGGTCCAGGTGATGTTGT | 189 |
| ***SLC6A2 (NET)*** | GTGCAGCCCGAGAACAAC | GCCGTTCTTGTAGCAGAGGT | 243 |
| ***SLC19A3 (THTR2)*** | CCTACCAATGCCCAAGAAAA | TTGGAACCACTGCACAAAAA | 235 |
| ***SLC29A4 (ENT4)*** | CGGCGGGCGTGATGATCTCT | CTAACAGGTGCAGCAGGAAC | 212 |
| ***MnSOD (SOD2)*** | TCTGTTGGTGTCCAAGGCTC | GTTCCTTGCAGTGGATCCTGA | 104 |
| ***HIF1a*** | CCTTCGATCAGTTGTCACCA | TGGGTAGGAGATGGAGATGC | 211 |
| ***HIF2a (EPAS)*** | AGCAGCTGGAGAGCAAGAAG | GCCACTGGGTATTGGATCTG | 150 |
| ***XO/XDH*** | CCTTGGCCTCTTCACCCTAG | TCAATGGGGATGCTGCCAAA | 108 |
| ***GP91PHOX (CYBB)*** | TGCCTTTGAGTGGTTTGCAG | TCATCCCAGCCAGTGAGGTA | 108 |
| ***P22PHOX (CYBA)*** | GAGCAGTGGACGCCCATC | TCCTCCTCGCTGGGCTTC | 128 |
| ***P47PHOX (NCF-1)*** | AGACGCAGCGCTCTAAACC | AGACGCCAGCTTCCGCTT | 101 |
| ***P67PHOX (NCF-2)*** | TGTTCCCATGCCCTACACAC | GCCGGAGCTCCAGTTTCTTA | 119 |
| ***TFAM*** | CAGCATGCTAAAGAGGACGA | TTGTGCGACGTAGAAGATCC | 100 |
| ***PGC1a*** | CAGGGGCAGATTTGTTCTTC | CGTCGTCAAAAACAGCTTGA | 172 |
| ***NRF1*** | GAGTGACCCAAACCGAACAT | TCAGCTGCTGTGGAGTTGAG | 181 |
| ***NRF2*** | GCTGCTCAGAATTGCAGAAA | TTTGCTGCAGGGAGTATTCA | 229 |
| ***COX1*** | AGACCAAACCTACGCCAAAA | AGCGAAGGCTTCTCAAATCA | 217 |
| ***IGFR1*** | GGAGCCAGAGAACATGGAGA | GCGTAAGGCTGTCTCTCGTC | 156 |
| ***B2M*** | ACTCACGTCATCCAGCAGAG | CACGGCAGGCATACTCATCT | 216 |

Supplementary Table 4: Primer sequences used in study
