# Supplementary material for: Impact of Metformin Treatment on Human Placental Energy Production and Oxidative Stress
Source: Front Cell Dev Biol. 2022 Jun 17;10:935403. doi: 10.3389/fcell.2022.935403 (PMC9247405; doi:10.3389/fcell.2022.935403)

Supplementary Figure 1: Example of oxygen consumption rate changes in response to mitochondrial stress test from sample of primary trophoblast. Assay reagents were loaded onto the sensor cartridge: A) Oligomycin (2µM), B) FCCP (2µM) and C) ROT/AA (0.5µM).

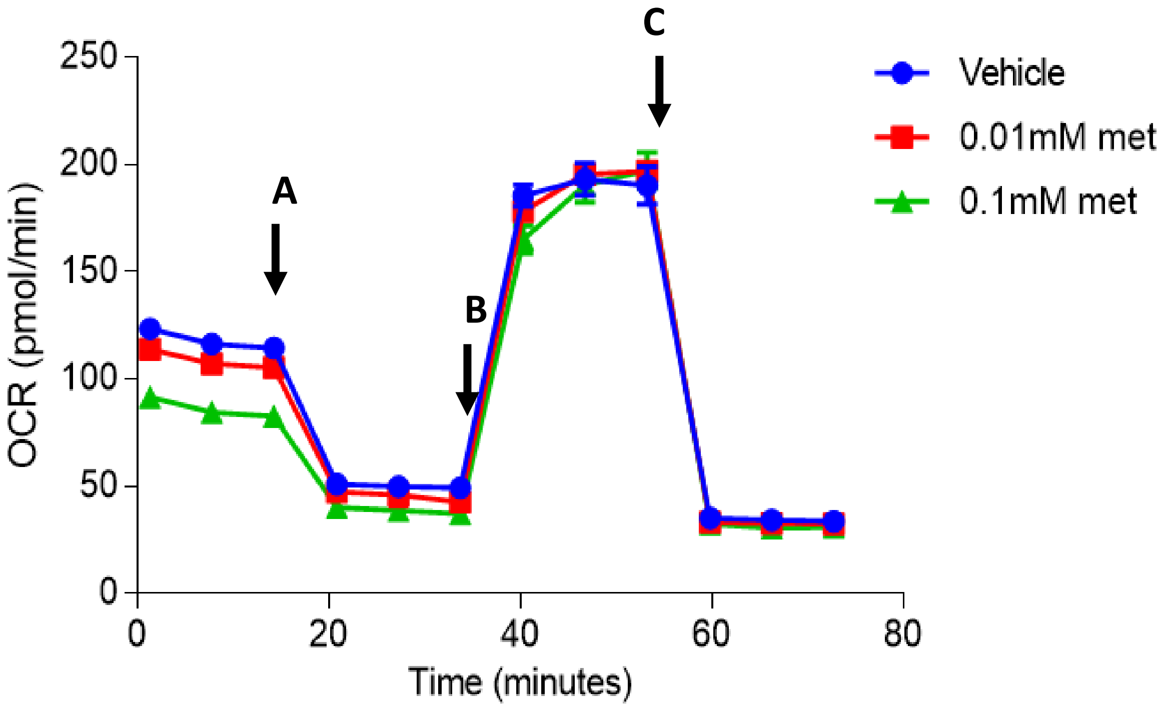

Supplement: Supplementary file 6 [file Image1.pdf]
